# Bis(azido-κ*N*)(di-2-pyridyl­amine-κ^2^ *N* ^2^,*N* ^2′^)palladium(II)

**DOI:** 10.1107/S1600536812013074

**Published:** 2012-03-31

**Authors:** Kwang Ha

**Affiliations:** a School of Applied Chemical Engineering, The Research Institute of Catalysis, Chonnam National University, Gwangju 500-757, Republic of Korea

## Abstract

In the title complex, [Pd(N_3_)_2_(C_10_H_9_N_3_)], the Pd^II^ ion is in a slightly distorted square-planar coordination environment. The ligator atoms comprise the two pyridine N atoms of the chelating di-2-pyridyl­amine (dpa) ligand and two N atoms from two azide anions. The dpa ligand coordinates the Pd atom in a boat conformation, the dihedral angle between the pyridine rings being 24.4 (1)°. The pyridine rings are somewhat inclined to the least-squares plane of the PdN_4_ unit, making dihedral angles of 29.02 (9) and 26.47 (9)°. The azide ligands are slightly bent, with N—N—N angles of 173.0 (4) and 174.2 (4)°. In the crystal, mol­ecules are connected by N—H⋯N and C—H⋯N hydrogen bonds, forming chains along the *c* axis. When viewed down the *b* axis, successive chains are stacked in opposite directions. Intra­molecular C—H⋯N hydrogen bonds are also observed.

## Related literature

For the crystal structures of the related Pd^II^ complexes [Pd*X*_2_(dpa)] (*X* = Cl or Br), see: Rauterkus *et al.* (2003 ▶); Yao *et al.* (2003 ▶).
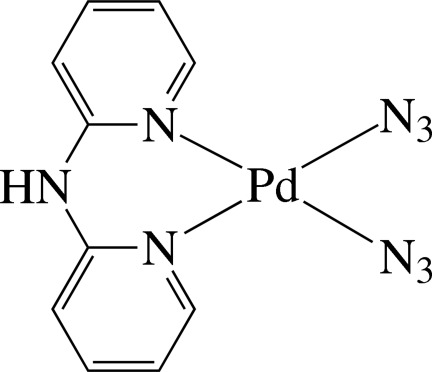


## Experimental

### 

#### Crystal data

[Pd(N_3_)_2_(C_10_H_9_N_3_)]
*M* *_r_* = 361.66
Monoclinic, 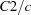

*a* = 17.5552 (15) Å
*b* = 6.9773 (6) Å
*c* = 19.6654 (17) Å
β = 99.206 (2)°
*V* = 2377.7 (4) Å^3^
*Z* = 8
Mo *K*α radiation
μ = 1.57 mm^−1^
*T* = 200 K
0.20 × 0.14 × 0.09 mm

#### Data collection

Bruker SMART 1000 CCD diffractometer
Absorption correction: multi-scan (*SADABS*; Bruker, 2000 ▶) *T*_min_ = 0.901, *T*_max_ = 1.000
7041 measured reflections
2322 independent reflections
1751 reflections with *I* > 2σ(*I*)
*R*_int_ = 0.029

#### Refinement

*R*[*F* ^2^ > 2σ(*F* ^2^)] = 0.029
*wR*(*F* ^2^) = 0.073
*S* = 1.06
2322 reflections
181 parameters
H-atom parameters constrained
Δρ_max_ = 0.71 e Å^−3^
Δρ_min_ = −0.41 e Å^−3^

### 

Data collection: *SMART* (Bruker, 2000 ▶); cell refinement: *SAINT* (Bruker, 2000 ▶); data reduction: *SAINT*; program(s) used to solve structure: *SHELXS97* (Sheldrick, 2008 ▶); program(s) used to refine structure: *SHELXL97* (Sheldrick, 2008 ▶); molecular graphics: *ORTEP-3* (Farrugia, 1997 ▶) and *PLATON* (Spek, 2009 ▶); software used to prepare material for publication: *SHELXL97*.

## Supplementary Material

Crystal structure: contains datablock(s) global. DOI: 10.1107/S1600536812013074/fj2539sup1.cif

Additional supplementary materials:  crystallographic information; 3D view; checkCIF report

## Figures and Tables

**Table 1:** Selected bond lengths (Å)

Pd1—N4	2.001 (3)
Pd1—N7	2.018 (3)
Pd1—N1	2.040 (3)
Pd1—N3	2.046 (3)

**Table 2:** Hydrogen-bond geometry (Å, °)

*D*—H⋯*A*	*D*—H	H⋯*A*	*D*⋯*A*	*D*—H⋯*A*
N2—H2*N*⋯N9^i^	0.92	2.31	3.208 (4)	165
C1—H1⋯N4	0.95	2.35	2.816 (5)	110
C4—H4⋯N6^i^	0.95	2.40	3.175 (5)	138
C10—H10⋯N7	0.95	2.35	2.861 (5)	113

Symmetry code: (i) 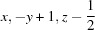.

**Fig. 1.**
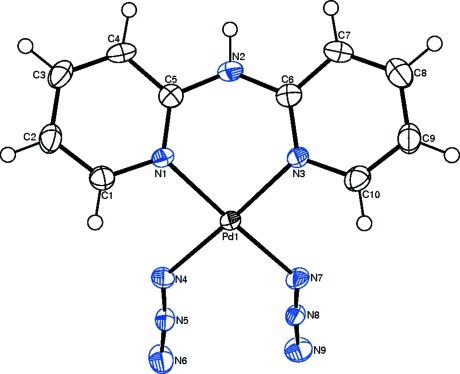
A structure detail of the title complex, with displacement ellipsoids drawn at the 50% probability level for non-H atoms.

**Fig. 2.**
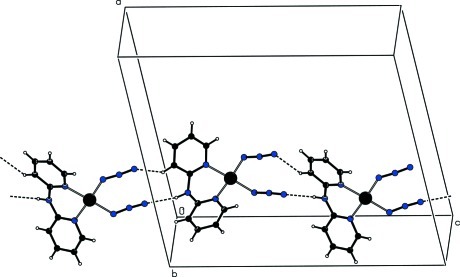
A partial view of the unit-cell contents of the title complex. Intermolecular N—H···N and C—H···N hydrogen-bond interactions are drawn with dashed lines.

## supplementary crystallographic information

### Comment

Crystal structures of Pd^II^ complexes with di-2-pyridylamine (dpa; C_10_H_9_N_3_) and halogen ions, [Pd*X*_2_(dpa)] (*X* = Cl or Br), have been reported previously (Rauterkus *et al.*, 2003; Yao *et al.*, 2003).

In the title complex, [Pd(N_3_)_2_(dpa)], the Pd^II^ ion is four-coordinated in a slightly distorted square-planar environment by the two pyridine N atoms of the chelating dpa ligand and two N atoms from two azide anions (Fig. 1). The dpa ligand coordinates the Pd atom in a boat conformation. The dihedral angle between the least-squares planes of the two pyridine rings is 24.4 (1)°. The pyridine rings are somewhat inclined to the least-squares plane of the PdN_4_ unit [maximum deviation = 0.016 (2) Å], making dihedral angles of 29.02 (9)° and 26.47 (9)°. The Pd—N(azide) and Pd—N(dpa) bond lengths are nearly equivalent [Pd—N: 2.001 (3)–2.046 (3) Å] (Table 1). The azide ligands are slightly bent with the bond angles of <N4—N5—N6 = 173.0 (4)° and <N7—N8—N9 = 174.2 (4)°. But, the N—N bond lengths of the ligands are almost equal [N—N: 1.146 (4)–1.212 (4) Å]. In the crystal, the complex molecules are connected by intermolecular N—H···N and C—H···N hydrogen bonds, forming chains along the *c* axis (Fig. 2 and Table 2). When viewed down the *b* axis, successive chains are stacked in opposite directions. Intramolecular C—H···N hydrogen bonds are also observed (Table 2).

### Experimental

To a solution of Na_2_PdCl_4_ (0.1451 g, 0.493 mmol) in MeOH (30 ml) were added NaN_3_ (0.3050 g, 4.692 mmol) and di-2-pyridylamine (0.0860 g, 0.502 mmol), and stirred for 5 h at room temperature. The formed precipitate was separated by filtration and washed with H_2_O and acetone, and dried at 50 °C, to give a yellow powder (0.1604 g). Crystals suitable for X-ray analysis were obtained by slow evaporation from a CH_3_CN/acetone solution.

### Refinement

Carbon-bound H atoms were positioned geometrically and allowed to ride on their respective parent atoms: C—H = 0.95 Å with *U*_iso_(H) = 1.2*U*_eq_(C). Nitrogen-bound H atom was located from the difference Fourier map then allowed to ride on its parent atom in the final cycles of refinement with N—H = 0.92 Å and *U*_iso_(H) = 1.5 *U*_eq_(N). The highest peak (0.71 e Å^-3^) and the deepest hole (-0.41 e Å^-3^) in the difference Fourier map are located 1.07 Å and 1.54 Å, respectively, from the Pd1 atom.

### Crystal data

**Table:**

[Pd(N_3_)_2_(C_10_H_9_N_3_)]	*F*(000) = 1424
*M**_r_* = 361.66	*D*_x_ = 2.021 Mg m^−^^3^
Monoclinic, *C*2/*c*	Mo *K*α radiation, λ = 0.71073 Å
Hall symbol: -C 2yc	Cell parameters from 3353 reflections
*a* = 17.5552 (15) Å	θ = 2.4–26.0°
*b* = 6.9773 (6) Å	µ = 1.57 mm^−^^1^
*c* = 19.6654 (17) Å	*T* = 200 K
β = 99.206 (2)°	Block, yellow
*V* = 2377.7 (4) Å^3^	0.20 × 0.14 × 0.09 mm
*Z* = 8	

### Data collection

**Table:**

Bruker SMART 1000 CCD diffractometer	2322 independent reflections
Radiation source: fine-focus sealed tube	1751 reflections with *I* > 2σ(*I*)
Graphite monochromator	*R*_int_ = 0.029
φ and ω scans	θ_max_ = 26.0°, θ_min_ = 2.1°
Absorption correction: multi-scan (*SADABS*; Bruker, 2000)	*h* = −21→17
*T*_min_ = 0.901, *T*_max_ = 1.000	*k* = −8→8
7041 measured reflections	*l* = −20→24

### Refinement

**Table:**

Refinement on *F*^2^	Primary atom site location: structure-invariant direct methods
Least-squares matrix: full	Secondary atom site location: difference Fourier map
*R*[*F*^2^ > 2σ(*F*^2^)] = 0.029	Hydrogen site location: inferred from neighbouring sites
*wR*(*F*^2^) = 0.073	H-atom parameters constrained
*S* = 1.06	*w* = 1/[σ^2^(*F*_o_^2^) + (0.0309*P*)^2^ + 3.0994*P*] where *P* = (*F*_o_^2^ + 2*F*_c_^2^)/3
2322 reflections	(Δ/σ)_max_ = 0.001
181 parameters	Δρ_max_ = 0.71 e Å^−^^3^
0 restraints	Δρ_min_ = −0.41 e Å^−^^3^

### Special details

**Table:**

Geometry. All e.s.d.'s (except the e.s.d. in the dihedral angle between two l.s. planes) are estimated using the full covariance matrix. The cell e.s.d.'s are taken into account individually in the estimation of e.s.d.'s in distances, angles and torsion angles; correlations between e.s.d.'s in cell parameters are only used when they are defined by crystal symmetry. An approximate (isotropic) treatment of cell e.s.d.'s is used for estimating e.s.d.'s involving l.s. planes.
Refinement. Refinement of *F*^2^ against ALL reflections. The weighted *R*-factor *wR* and goodness of fit *S* are based on *F*^2^, conventional *R*-factors *R* are based on *F*, with *F* set to zero for negative *F*^2^. The threshold expression of *F*^2^ > σ(*F*^2^) is used only for calculating *R*-factors(gt) *etc*. and is not relevant to the choice of reflections for refinement. *R*-factors based on *F*^2^ are statistically about twice as large as those based on *F*, and *R*- factors based on ALL data will be even larger.

### Fractional atomic coordinates and isotropic or equivalent isotropic displacement parameters (Å^2^)

**Table:**

	*x*	*y*	*z*	*U*_iso_*/*U*_eq_	
Pd1	0.251934 (14)	0.29162 (4)	0.249235 (13)	0.02012 (11)	
N1	0.31748 (16)	0.2924 (4)	0.17242 (15)	0.0204 (6)	
N2	0.21863 (17)	0.4333 (4)	0.09167 (16)	0.0283 (8)	
H2N	0.2104	0.4969	0.0502	0.042*	
N3	0.15471 (16)	0.2919 (4)	0.17634 (15)	0.0216 (7)	
N4	0.35049 (17)	0.2861 (5)	0.31632 (16)	0.0299 (8)	
N5	0.36249 (16)	0.3271 (4)	0.37610 (17)	0.0266 (7)	
N6	0.38160 (18)	0.3598 (6)	0.43357 (18)	0.0409 (9)	
N7	0.18386 (17)	0.2944 (5)	0.32278 (15)	0.0266 (7)	
N8	0.19687 (16)	0.3593 (5)	0.38063 (16)	0.0258 (7)	
N9	0.20315 (19)	0.4148 (5)	0.43611 (17)	0.0386 (9)	
C1	0.3921 (2)	0.2266 (6)	0.18546 (19)	0.0285 (9)	
H1	0.4083	0.1574	0.2269	0.034*	
C2	0.4441 (2)	0.2556 (6)	0.1421 (2)	0.0334 (10)	
H2	0.4952	0.2077	0.1530	0.040*	
C3	0.4206 (2)	0.3575 (6)	0.0812 (2)	0.0336 (9)	
H3	0.4564	0.3859	0.0512	0.040*	
C4	0.3451 (2)	0.4161 (6)	0.06515 (19)	0.0293 (9)	
H4	0.3275	0.4816	0.0233	0.035*	
C5	0.2950 (2)	0.3771 (5)	0.11182 (18)	0.0239 (8)	
C6	0.1522 (2)	0.3672 (5)	0.11384 (18)	0.0237 (8)	
C7	0.0833 (2)	0.3866 (6)	0.06780 (19)	0.0306 (9)	
H7	0.0829	0.4454	0.0242	0.037*	
C8	0.0162 (2)	0.3195 (6)	0.0865 (2)	0.0375 (10)	
H8	−0.0312	0.3297	0.0557	0.045*	
C9	0.0183 (2)	0.2366 (6)	0.1508 (2)	0.0349 (10)	
H9	−0.0275	0.1898	0.1649	0.042*	
C10	0.0877 (2)	0.2234 (6)	0.1935 (2)	0.0309 (9)	
H10	0.0893	0.1636	0.2371	0.037*	

### Atomic displacement parameters (Å^2^)

**Table:**

	*U*^11^	*U*^22^	*U*^33^	*U*^12^	*U*^13^	*U*^23^
Pd1	0.02099 (16)	0.02298 (17)	0.01673 (16)	0.00000 (12)	0.00408 (10)	0.00056 (12)
N1	0.0239 (15)	0.0216 (16)	0.0162 (16)	−0.0020 (12)	0.0047 (12)	−0.0003 (13)
N2	0.0321 (18)	0.0315 (19)	0.0216 (18)	−0.0010 (14)	0.0052 (14)	0.0068 (14)
N3	0.0223 (15)	0.0254 (17)	0.0177 (16)	0.0004 (12)	0.0052 (12)	−0.0014 (13)
N4	0.0252 (17)	0.048 (2)	0.0152 (17)	0.0007 (15)	0.0007 (13)	−0.0011 (15)
N5	0.0199 (16)	0.0304 (19)	0.030 (2)	0.0012 (13)	0.0061 (14)	0.0027 (15)
N6	0.0316 (19)	0.064 (3)	0.026 (2)	0.0045 (18)	0.0017 (15)	−0.0128 (19)
N7	0.0278 (17)	0.036 (2)	0.0168 (17)	−0.0026 (14)	0.0066 (13)	−0.0029 (14)
N8	0.0219 (16)	0.0279 (18)	0.029 (2)	0.0031 (13)	0.0086 (13)	0.0072 (15)
N9	0.043 (2)	0.055 (3)	0.0188 (19)	0.0027 (17)	0.0069 (15)	−0.0075 (17)
C1	0.028 (2)	0.033 (2)	0.024 (2)	0.0014 (17)	0.0032 (16)	−0.0021 (17)
C2	0.025 (2)	0.044 (3)	0.033 (2)	0.0013 (17)	0.0092 (17)	−0.008 (2)
C3	0.031 (2)	0.044 (3)	0.028 (2)	−0.0066 (19)	0.0133 (17)	−0.009 (2)
C4	0.041 (2)	0.030 (2)	0.016 (2)	−0.0032 (17)	0.0063 (16)	0.0008 (16)
C5	0.0278 (19)	0.0212 (19)	0.023 (2)	−0.0016 (16)	0.0038 (15)	−0.0058 (17)
C6	0.032 (2)	0.0182 (19)	0.022 (2)	0.0030 (16)	0.0066 (15)	−0.0053 (16)
C7	0.038 (2)	0.030 (2)	0.022 (2)	0.0006 (19)	−0.0013 (16)	0.0027 (18)
C8	0.030 (2)	0.043 (3)	0.037 (3)	0.0045 (18)	−0.0019 (18)	−0.005 (2)
C9	0.024 (2)	0.044 (3)	0.037 (3)	−0.0038 (18)	0.0063 (17)	−0.005 (2)
C10	0.033 (2)	0.036 (2)	0.025 (2)	−0.0019 (18)	0.0094 (17)	−0.0033 (18)

### Geometric parameters (Å, º)

**Table:**

Pd1—N4	2.001 (3)	C1—H1	0.9500
Pd1—N7	2.018 (3)	C2—C3	1.397 (5)
Pd1—N1	2.040 (3)	C2—H2	0.9500
Pd1—N3	2.046 (3)	C3—C4	1.374 (5)
N1—C5	1.332 (4)	C3—H3	0.9500
N1—C1	1.373 (4)	C4—C5	1.396 (5)
N2—C6	1.387 (4)	C4—H4	0.9500
N2—C5	1.393 (4)	C6—C7	1.398 (5)
N2—H2N	0.9200	C7—C8	1.370 (5)
N3—C6	1.331 (4)	C7—H7	0.9500
N3—C10	1.361 (5)	C8—C9	1.386 (6)
N4—N5	1.195 (4)	C8—H8	0.9500
N5—N6	1.149 (4)	C9—C10	1.368 (5)
N7—N8	1.212 (4)	C9—H9	0.9500
N8—N9	1.146 (4)	C10—H10	0.9500
C1—C2	1.360 (5)		
			
N4—Pd1—N7	94.38 (12)	C4—C3—C2	119.3 (4)
N4—Pd1—N1	87.57 (12)	C4—C3—H3	120.3
N7—Pd1—N1	177.94 (11)	C2—C3—H3	120.3
N4—Pd1—N3	176.68 (11)	C3—C4—C5	118.5 (4)
N7—Pd1—N3	88.79 (12)	C3—C4—H4	120.8
N1—Pd1—N3	89.28 (11)	C5—C4—H4	120.8
C5—N1—C1	116.9 (3)	N1—C5—N2	120.9 (3)
C5—N1—Pd1	122.9 (2)	N1—C5—C4	123.2 (3)
C1—N1—Pd1	119.7 (2)	N2—C5—C4	116.0 (3)
C6—N2—C5	129.5 (3)	N3—C6—N2	121.1 (3)
C6—N2—H2N	114.8	N3—C6—C7	122.2 (3)
C5—N2—H2N	113.3	N2—C6—C7	116.6 (3)
C6—N3—C10	117.8 (3)	C8—C7—C6	119.0 (4)
C6—N3—Pd1	123.2 (2)	C8—C7—H7	120.5
C10—N3—Pd1	118.9 (2)	C6—C7—H7	120.5
N5—N4—Pd1	129.9 (3)	C7—C8—C9	119.2 (4)
N6—N5—N4	173.0 (4)	C7—C8—H8	120.4
N8—N7—Pd1	129.2 (2)	C9—C8—H8	120.4
N9—N8—N7	174.2 (4)	C10—C9—C8	118.7 (4)
C2—C1—N1	123.3 (4)	C10—C9—H9	120.7
C2—C1—H1	118.4	C8—C9—H9	120.7
N1—C1—H1	118.4	N3—C10—C9	123.0 (4)
C1—C2—C3	118.6 (4)	N3—C10—H10	118.5
C1—C2—H2	120.7	C9—C10—H10	118.5
C3—C2—H2	120.7		
			
N4—Pd1—N1—C5	−149.3 (3)	C1—N1—C5—C4	−5.9 (5)
N3—Pd1—N1—C5	31.7 (3)	Pd1—N1—C5—C4	165.2 (3)
N4—Pd1—N1—C1	21.6 (3)	C6—N2—C5—N1	−22.4 (6)
N3—Pd1—N1—C1	−157.4 (3)	C6—N2—C5—C4	157.9 (4)
N7—Pd1—N3—C6	151.4 (3)	C3—C4—C5—N1	2.7 (6)
N1—Pd1—N3—C6	−27.9 (3)	C3—C4—C5—N2	−177.7 (3)
N7—Pd1—N3—C10	−24.3 (3)	C10—N3—C6—N2	−177.5 (3)
N1—Pd1—N3—C10	156.4 (3)	Pd1—N3—C6—N2	6.8 (5)
N7—Pd1—N4—N5	−18.7 (4)	C10—N3—C6—C7	3.5 (5)
N1—Pd1—N4—N5	160.7 (4)	Pd1—N3—C6—C7	−172.2 (3)
N4—Pd1—N7—N8	29.4 (3)	C5—N2—C6—N3	26.7 (6)
N3—Pd1—N7—N8	−151.6 (3)	C5—N2—C6—C7	−154.3 (4)
C5—N1—C1—C2	4.6 (5)	N3—C6—C7—C8	−2.5 (6)
Pd1—N1—C1—C2	−166.9 (3)	N2—C6—C7—C8	178.5 (4)
N1—C1—C2—C3	0.1 (6)	C6—C7—C8—C9	0.9 (6)
C1—C2—C3—C4	−3.5 (6)	C7—C8—C9—C10	−0.4 (6)
C2—C3—C4—C5	2.2 (6)	C6—N3—C10—C9	−3.1 (6)
C1—N1—C5—N2	174.4 (3)	Pd1—N3—C10—C9	172.8 (3)
Pd1—N1—C5—N2	−14.4 (5)	C8—C9—C10—N3	1.5 (6)

### Hydrogen-bond geometry (Å, º)

**Table:**

*D*—H···*A*	*D*—H	H···*A*	*D*···*A*	*D*—H···*A*
N2—H2*N*···N9^i^	0.92	2.31	3.208 (4)	165
C1—H1···N4	0.95	2.35	2.816 (5)	110
C4—H4···N6^i^	0.95	2.40	3.175 (5)	138
C10—H10···N7	0.95	2.35	2.861 (5)	113

Symmetry code: (i) *x*, −*y*+1, *z*−1/2.
